# Alternative to oral dicoumarin anticoagulants: Considerations in dental care

**DOI:** 10.4317/jced.51226

**Published:** 2013-12-01

**Authors:** Ana Mingarro-de-León, Begonya Chaveli-López

**Affiliations:** 1 Oral Medicine, Department of Stomatology, Valencia University, Valencia, Spain

## Abstract

Introduction: For over 50 years, vitamin K antagonists such as warfarin (Aldocumar®) and acenocoumarol (Sintrom®) have been the gold standard for reducing the risk of cerebrovascular events. In the last 5 years alternative anticoagulants have been evaluated that act directly upon a concrete target within the coagulation cascade, thereby affording a more predictable anticoagulant effect. The present study offers an update on the new oral anticoagulants and reviews the implications referred to the dental care of patients administered these substances. Material and methods: An exhaustive PubMed-Medline and Cochrane Library search was made of the main alternatives to conventional oral anticoagulation, covering those studies published in English and Spanish over the last 10 years. Specialized textbooks and pharmaceutical catalogs were also consulted. A total of 184 articles were identified, of which 76 met the inclusion criteria. Results: The new oral anticoagulants dabigatran, rivaroxaban and apixaban are safe and effective, and offer a series of advantages, including rapid action, no need for constant monitoring, few drug and food interactions, and a broad therapeutic margin. These drugs are expensive, however, and some lack a specific antidote, while others must be administered twice a day. Regarding the dental treatment of patients receiving these drugs, suspension or modification of the background medication is not required when performing invasive dental procedures, except where indicated by the prescribing physician. Conclusions: The new oral anticoagulants do not pose significantly greater risks than conventional oral anticoagulants when providing invasive dental treatment, and their suspension is not strictly required in such situations.

** Key words:**Dabigatran, rivaroxaban, apixaban, dental, hemostasis.

## Introduction

As a result of the aging of the population and the increase in life expectancy, the prevalence of chronic diseases, including heart disorders and cerebrovascular events, is growing (1). In order to prevent thromboembolic problems and infarction, these patients often receive anticoagulant treatment – the concrete indications of which include atrial fibrillation and other heart arrhythmias; venous thromboembolism (deep venous thrombosis, pulmonary embolism); acute coronary syndrome and myocardial infarction; pulmonary hypertension; and heart valve disease and valve prostheses (1,2).

In general terms, oral anticoagulants are effective and reliable, offering good tolerance, and rapid absorption after oral administration, with peak plasma concentrations being reached after one hour (3,4). In the United Kingdom, it has been estimated that about 300,000 people receive treatment with oral anticoagulants – the proportional number in Spain being approximately 250,000 patients. For decades, the drugs used in oral anticoagulation therapy have been the vitamin K antagonists (VKAs) [acenocoumarol (Sintrom®) and warfarin (Aldocumar®)], and in patients with special risks or contraindications to VKAs, antiplatelet medication has been used as an alternative (5). However, these anticoagulants may give rise to adverse effects and interactions with different drugs and foods. Furthermore, although the antithrombotic effects manifest after 48-72 hours, a decrease in coagulation factors is only observed after 5 days of therapy (6). The clinical management of these drug substances is therefore complicated by the need for close monitoring of their activity. These and other factors have limited the use of such medicines in routine clinical practice, and there has always been a need for new oral anticoagulant drugs offering easier handling characteristics, a better safety profile, and fewer drug interactions (7).

In this context, Haremberg et al. in the year 2008 (8) defined the ideal anticoagulant as a drug offering rapid onset of action and a short half-life (easy handling performance in the event of bleeding, without the need to add other anticoagulants); predictable pharmacokinetics (easier dosing); a predictable anticoagulant effect (fixed dose, without the need for monitoring); administration via the oral route (thereby facilitating the definition of new indications); metabolism not mediated by isoenzyme CYP2C9 or VCOR1 (i.e., without drug or food interactions); availability of an antidote (safety in the event of bleeding); and an adequate cost (thereby facilitating clinical development). In addition, the development of new anticoagulants should seek to offer a small molecular weight synthetic drug specifically and directly acting upon a single coagulation factor (Xa/IIa), with none of the known undesired effects of the current drugs, such as the coumarin derivatives (7,9,10). Accordingly, in the last 5 years, alternative anticoagulants (dabigatran, rivaroxaban and apixaban) have been evaluated that act directly upon a concrete target within the coagulation cascade, thereby affording a more predictable anticoagulant effect.

The present study offers an update on the new oral anticoagulants and reviews the implications referred to the dental care of patients administered these substances.

## Material and methods

An exhaustive PubMed-Medline and Cochrane Library search was made of the main alternatives to conventional oral anticoagulation. The key words used were “dabigatran”, “rivaroxaban” and “apixaban”, with the boolean operator «AND». We included studies published in English and Spanish over the last 10 years. Specialized textbooks and pharmaceutical catalogs were also consulted. A total of 184 articles were identified, of which 76 (68 literature reviews, 4 metaanalyses and systematic reviews, and 7 clinical trials) met the inclusion criteria. It should be noted that the search yielded only three studies on the new oral anticoagulants published in the dental literature.

## Coagulation cascade

The coagulation cascade was first described in the mid-1960s, based on in vitro experimental data, and comprises a series of steps through the so-called intrinsic and extrinsic coagulation pathways. The intrinsic and extrinsic pathways activate different coagulation factors and converge in a common pathway that leads to the conversion of factor X to activated factor Xa. The latter is a key element in the conversion of prothrombin (factor II) into thrombin, which in turn converts fibrinogen (factor I) into fibrin (1,11). However, it was soon seen that the two pathways do not operate independently of each other, and that the above described model is unable to explain the physiopathological processes occurring in the context of vascular damage (12).

**Figure 1:**
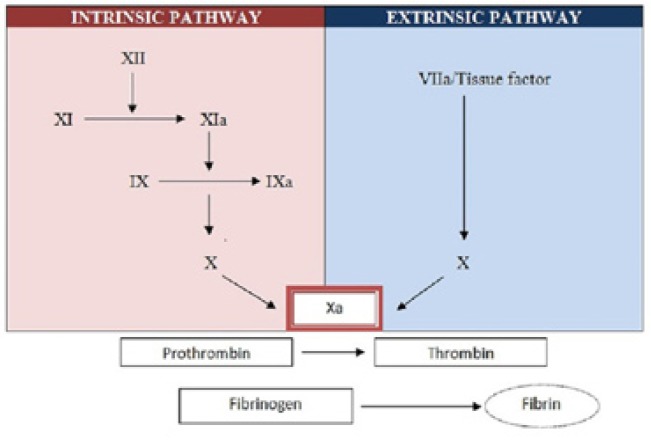
Classical blood coagulation cascade.

More recent studies demonstrated the importance of the cellular component in the coagulation process. According to the current model, coagulation comprises three interrelated phases. Initiation phase: this phase is mediated by the tissue factor (TF) producing cells. TF is a protein found in the membrane of different cells such as fibroblasts and monocytes, and gives rise to the generation of factors Xa and IXa, and to small amounts of thrombin that suffice to start the coagulation process. Amplification phase: in this phase the platelets are activated by the generated thrombin, and accumulate factors and cofactors at surface level, thereby facilitating the different enzymatic reactions. Lastly, in the propagation phase, proteases combine with the cofactors at platelet surface level, facilitating the production of large amounts of thrombin that in turn lead to the formation of fibrin and its polymerization to form a stable blood clot (Fig. 1) (12).

## Activity of the new oral anticoagulants

At present, the “new” oral anticoagulants, offering improved possibilities for clinical use, can be classified as direct thrombin inhibitors or oral activated factor X inhibitors (4,7,9). The anticoagulants belonging to the first group are competitive thrombin (factor IIa) inhibitors that avoid the formation of fibrin from fibrinogen, independently of the presence of antithrombin, and can inactivate both the free (soluble) form of thrombin and thrombin bound to fibrin. This group of drugs includes ximelagatran and dabigatran (13). The anticoagulants belonging to the second group in turn bind to the active site of factor Xa in both its soluble form and bound to the prothrombinase complex, thereby blocking its interaction with thrombin. A number of drugs are under study in this group, though rivaroxaban and apixaban are the molecules in the most advanced stages of development (5,7,9,14).

- Dabigatran etexilate (Pradaxa®)

Dabigatran etexilate is a direct thrombin inhibitor that prevents the conversion of fibrinogen to fibrin (10). Its main indication is in programmed (elective) total hip or knee replacement surgery. The drug is also indicated in the prevention of stroke and systemic embolic disorders in adults with non-valvular atrial fibrillation (10,15). It is administered twice a day, and its bioavailability after oral dosing is 6-7%. Dabigatran etexilate is very fast acting and reaches peak plasma concentrations between 1-6 hours after administration, with a half-life of 14-17 hours. Most of the drug is eliminated through the kidneys (80%), and 20% is excreted in bile. Routine monitoring is not required (10,15,16). No specific antidote to the effects of dabigatran etexilate has been developed to date (14,16-18). Interactions have been observed with rifampicin, amiodarone, and P-glycoprotein-P inducers / inhibitors (10). The most frequent adverse effects (15%) are symptoms of gastritis, including dyspepsia, abdominal pain, and epigastric discomfort (19).

- Rivaroxaban (Xarelto®)

Rivaroxaban is an oxazolidinone derivative that exerts a direct inhibitory effect upon factor Xa (20), thereby blocking the transformation of prothrombin into thrombin, and thus ultimately inhibiting blood clot formation (16). It is indicated in the prevention of venous thromboembolism in adults subjected to elective hip or knee replacement surgery, and in the treatment of deep venous thrombosis (DVT) and pulmonary embolism following DVT in adult patients. The drug is also indicated in the prevention of stroke and systemic embolic disorders in adults with non-valvular atrial fibrillation who present one or more risk factors (congestive heart failure, hypertension, age ≥ 75 years, diabetes mellitus, previous stroke or transient ischemic attack episodes) (10,20,21). Rivaroxaban shows high bioavailability following oral administration, and a rapid onset of action (22). The peak plasma concentrations are reached 1.5-2 hours after administration. The plasma half-life is 5-9 hours in young individuals and 12-33 hours in patients > 75 years of age (10,23). The drug is eliminated in urine and bile (20). Rivaroxaban shows few interactions with commonly used drugs such as aspirin, clopidogrel, digoxin or naproxen (1). There have been reports of dyspepsia following administration of the drug – this being its most frequent non-hematological adverse effect. In the same way as the rest of the direct action oral anticoagulants, rivaroxaban has no specific antidote (10). However, although protamine sulfate is not effective, some studies suggest that recombinant factor VIIa (Novo Siete®) or active concentrated prothrombin complex can revert the anticoagulant effect (24). No monitoring is required with the use of rivaroxaban.

- Apixaban (Eliquis®)

Apixaban is a potent and reversible factor Xa inhibitor with the same therapeutic indications as the above described drugs (11,21). Its oral bioavailability is close to 60%, and the peak plasma concentrations are reached 1-3 hours after administration, with an elimination half-life of about 12 hours (11). The drug is fundamentally excreted in bile (16). In the same way as dabigatran, apixaban is administered twice a day and has no confirmed specific antidote – though in extreme cases we can use rFVIIa, rFXa and activated prothrombin complexes. However, in situations of normal bleeding, it suffices to postpone a dose or suspend the medication, though further studies are needed in this respect (13,25-27). Combined administration with potent P-glycoprotein and CYP3A4 inhibitors is contraindicated (11). Apixaban is the most recently marketed direct anticoagulant (January 2013) (1).

 Table 1 describes the main differences between the vitamin K antagonists (VKAs) and the new oral anticoagulants.

**Table 1:**
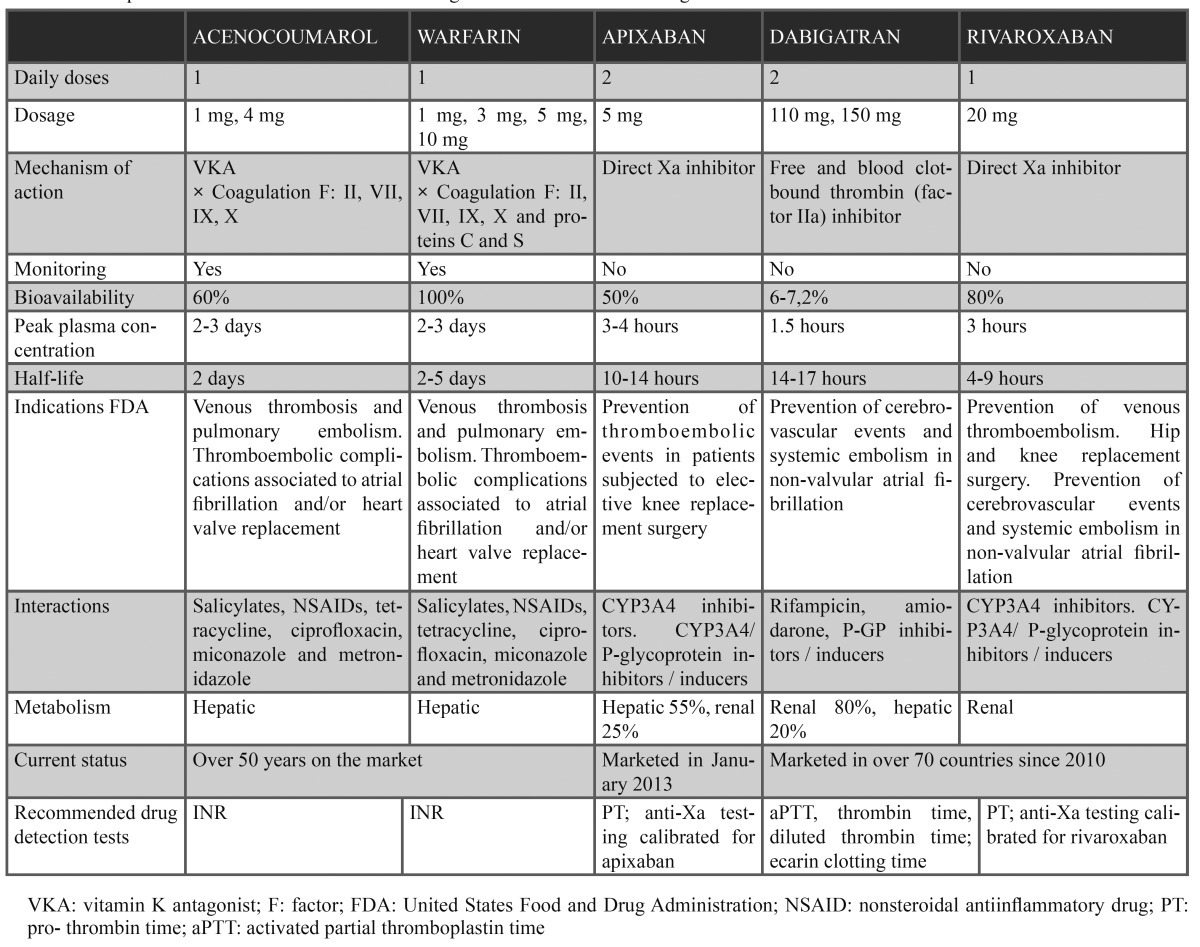
Principal characteristics of the vitamin K antagonists and new oral anticoagulants.

## Advantages and inconveniences: new anticoagulants versus vitamin K antagonists

The new direct oral anticoagulants appear to offer a series of advantages with respect to the classical vitamin K antagonists (VKAs) ( Table 2). VKAs are slow in acting, and patients requiring immediate anticoagulation therefore need bridging therapy with a rapid acting agent such as heparin. VKAs also present numerous food and drug interactions, making it necessary to adopt measures of caution referred to diet and especially to changes in concomitant medication. Likewise, the classical drugs have a narrow therapeutic margin or window, which implies an unpredictable anticoagulant effect, with the need for regular coagulation controls and dose adjustments in order to keep the international normalized ratio (INR) within normal limits (16,28).

**Table 2:**
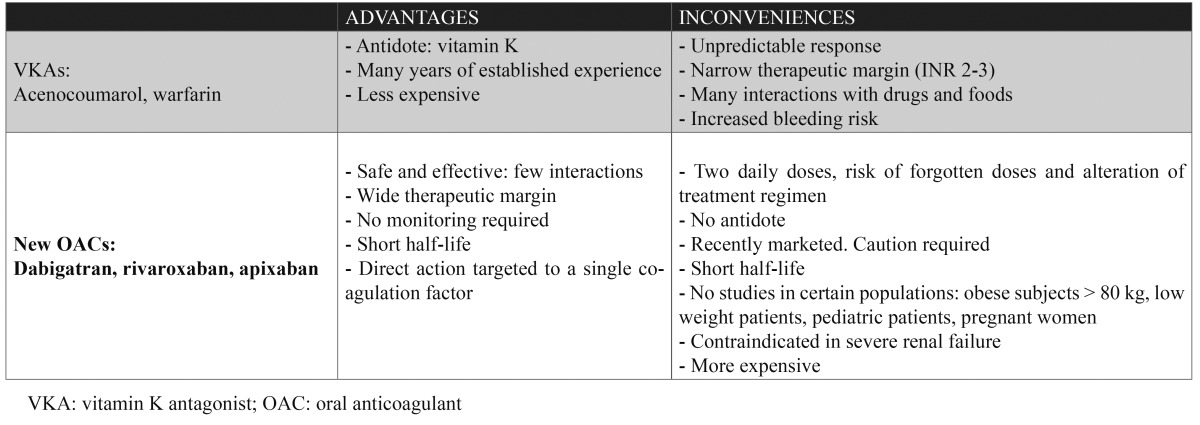
Advantages and inconveniences of the vitamin K antagonists and new oral anticoagulants.

The main difference between the two groups of drugs is their mechanism of action. In effect, while the classical drugs block d-carboxylation of the vitamin K-dependent coagulation factors (II, VII, IX, X), the new anticoagulants inhibit the coagulation factors directly, thereby ensuring a safer and more predictable response (1,6,7,18). Another important difference is that the new anticoagulants do not require routine monitoring (18). As regards cost-effectiveness, although the long-term repercussions are not clear (14), a RE-LY study in Navarra (Spain) conducted in 2011 reported that obviation of the need to control INR would save millions of euros in public spending over the long term (29). However, knowing the level of anticoagulation may prove necessary in many situations, including emergencies, the evaluation of treatment compliance, dose confirmation, and for reassuring the patient. In this context, sensitive tests have been developed that can give us an idea of the anticoagulation status of the patient ( Table 1) (11).

Despite the abovementioned advantages, the clinical experience gained with the new oral anticoagulants is still limited, and there are also potential inconveniences such as the lack of a direct antidote or of studies conducted in specific patient population, e.g., obese individuals, pregnant women, pediatric or low-weight patients, etc. (18,20,30,31). The fact that the new drugs have a relatively short half-life compared with the VKAs is an advantage when it comes to suspending anticoagulation in the context of programmed surgery, though it is also an inconvenience when patients forget to take a dose, since such omission leaves them unprotected until the next dose is taken (14,31).

## Considerations in dental care

Our review of the literature only yielded three studies (reviews) on the new oral anticoagulants in the dental care setting (1,10,15). A summarized account of the considerations in dental care among patients receiving the new oral anticoagulants is provided below.

There has been controversy for years regarding the suspension or alternation of anticoagulant therapy when planning invasive dental treatments. It is well known that there is a risk of embolism after suspending antithrombotic medication. For this reason, physicians tend to be conservative and avoid the suspension of antithrombotic medication (particularly with VKAs), provided there is no clear contraindication to suspension (6,22,26,32-34).

Regarding the new direct anticoagulants, the limited randomized clinical trials conducted to date do not allow us to establish a management protocol. Nevertheless, the results based on evidence corresponding to the classical anticoagulants, and the existing reviews on the new drugs, allow us to establish a series of guidelines.

Two recommendations can be established, depending on the bleeding risk of the planned dental treatment:

- Minor bleeding risk (simple extractions < 3, surgery lasting less than 45 minutes) (35): Suspension of the direct acting anticoagulant does not seem necessary in these cases. Hemostasis is to be facilitated, with local measures designed to help healing and minimize the risk of post-extraction bleeding (10,15,35).

- Major bleeding risk (multiple extractions > 3, surgery lasting more than 45 minutes, head and neck cancer surgery) (35): Operations of this kind can give rise to potentially life-threatening bleeding complications. Therefore, and depending on the type of operation, the existence of renal failure, and the risk of hemostatic alterations, the medication should be suspended for a number of days before the operation is carried out (33). Firriolo et al. (10) and Little (15) recommend suspending the anticoagulant 24 hours before the operation, followed by reintroduction once hemostatic control has been achieved. In contrast, other authors such as Spyropoulus et al. (35), recommend suspension 2-3 days before general surgery, followed by reintroduction of the medication after 24 hours.

Another factor to be taken into account in the management of these patients is renal function. In this sense, some authors such as Van Ryn et al. (36) offer recommendations depending on the creatinine clearance values of the patient ( Table 3).

**Table 3:**
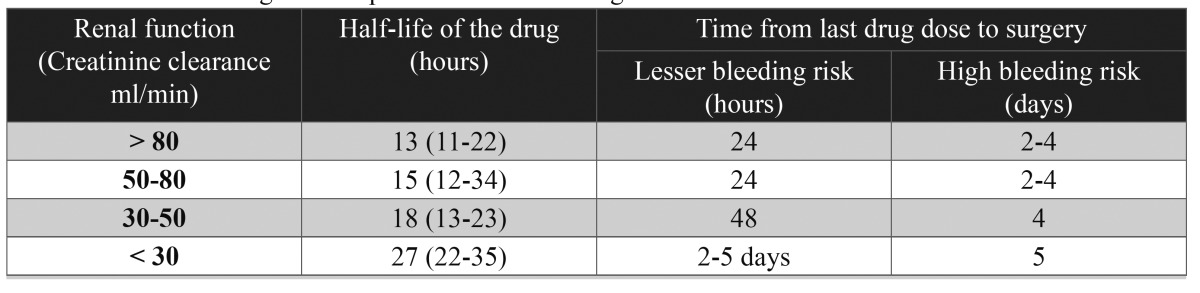
Direct anticoagulant suspension time according to creatinine clearance value.

Management must be optimized from preoperative workup to the postoperative period in all patients subjected to antithrombotic treatment. Surgery should be performed in the morning and at the start of the week in order to guarantee enough time to adequately deal with any possible complications (6,10,32,37,38). Good soft tissue management is essential in this sense, in order to avoid excessive traumatism of the surgical zone.

The main complication we can find is the lack of an antidote (30), which may pose a risk in the case of heavy bleeding as a result of dental surgery. Nevertheless, there are a number of lines of research, including the use of prothrombin complex concentrates (PCCs), which could neutralize the effect of rivaroxaban; the administration of recombinant factor VIIa; or the use of dialysis for neutralizing the effect of dabigatran. These options must be viewed with caution, however, since further studies are needed in order to confirm their efficacy (11,31,33,34). It is therefore important to use local hemostatic measures to help ensure good bleeding control (32). Supplementary measures have also been recommended, such as the use of absorbable hemostatic dressings in the form of oxidized cellulose (Surgicel®), collagen sponges (Haemocollagel®), or reabsorbable gelatin sponges (Spongostan®). In order to avoid postoperative bleeding, it is advisable to suture the extraction socket and apply compressive dressings impregnated with tranexamic acid (1).

## Conclusions

The introduction of the new direct action oral anticoagulants poses a number of challenges in dental surgery. The lack of an antidote in the event of postoperative bleeding requires maximum caution when using these drugs. Further randomized studies are needed to establish the efficacy and safety of dental treatment in patients receiving these new anticoagulants.
